# A wideband CPW slot antenna by using conductor-backed coplanar feeding

**DOI:** 10.1016/j.heliyon.2024.e40628

**Published:** 2024-11-23

**Authors:** Yulei Xue, Ruiqiang Yan, Weiping Huang, Yanchen Dong

**Affiliations:** a School of Information Science and Engineering, Shandong University, Qingdao, 266237, China; b School of Physical Science and Technology, Ningbo University, Ningbo, 315211, China; c School of Microelectronics, Shandong University, Jinan, 250100, China

**Keywords:** Rhomboid slot antenna, Wideband, Conductor-backed coplanar waveguide, Radiation pattern

## Abstract

In this paper, a new wideband coplanar waveguide (CPW) rhomboid slot antenna by using conductor-backed coplanar waveguide (CBCPW) feeding, which works at 4.6 GHz with a relative impedance bandwidth of about 55.6 %, and a gain of more than 5.5dBi from 4 to 6 GHz, has been proposed, and the performance of a CPW circular slot antenna and a CPW rectangular slot antenna are also used for comparing with the proposed antenna. It is demonstrated that the CPW rhomboid slot antenna has wider impedance bandwidth, higher gain and better radiation pattern compared with the circular and rectangular slot antennas, meanwhile, low profile has also realized. The CPW rhomboid slot antenna with CBCPW feeding has been fabricated and measured, and the design has been verified.

## 1 Introduction

The communication capacity of modern wireless communication system is closely related to the bandwidth of antenna. With the rapid growth of wireless data disposing, higher requirements are put forward for the capacity of communication system. Nowadays, with the fast development of communication technology, more and more information need to be transmitted and received, which make broadband technology be more and more popular. Wideband antenna is the essential component for broadband communication. Wideband antenna has been widely used in many kinds of communication systems such as base station et al. In many small mobile communication systems, low profile is also required. Wide-slot antenna has the advantages of wide band, low cost, good omnidirectional or bidirectional pattern.

As one of the main components of RF front-end of transceiver, antenna surface waves need to be minimized in the design. Slot antennas [[1], [2], [3], [4], [5], [6], [7], [8], [9], [10], [11], [12], [13], [14], [15], [16], [17], [18], [19], [20]] not only can reduce antenna size, increase antenna bandwidth, but also can reduce the surface waves because the ground area reduction can effectively suppress the surface wave propagation and flat pattern between the metal plate [2]. In the past years, slot antennas with U-shaped slot [3], V-shaped slot [4], E-shaped slot [5] and H-shaped slot [7], have been developed. It is seen that different slot brings different impedance bandwidth, and in order to increase the bandwidth, more complicated slots have been developed. In Refs. [6,8], antennas bandwidth are extended by inserting two inverted L-shaped branches into the square slot of the ground plane, and inserting an inverted L-shaped branch in a pair of spiral slots, respectively. In Ref. [11], a U-slot loaded E-shaped patch antenna is designed to extend the impedance bandwidth to 64.7 %, and in Ref. [12], multi-slotted antenna is designed for multi-functional applications. In Ref. [19], parametric analysis of coplanar waveguide (CPW) feed planar antenna for frequencies from 2.6 GHz to 13.6 GHz which covers the ultra-wideband from 3.1 to 10.6 GHz and the X-band from 8 to 12 GHz applications have been researched, and antenna examples with gain of about 7.3/7.5 dBi and a fractional bandwidth of about 93 % have been provided, however, the design lacks of experiment. In Ref. [20], multi-hexagonal slots antenna with defected ground structure is designed, which realizes a working frequency band from 2.75 GHz to 4.94 GHz, and a maximum gain of 4.3dBi, while a RLC equivalent circuit model is also proposed. It is seen that more hexagonal slots bring wider bandwidth and higher antenna gain, however, which introduce more complex antenna structure and larger antenna size. It is noted that most reported antennas are microstrip ones, and in many slot antenna designs, the impedance bandwidth is less than 50 %, while the antenna structures are complicated. There are few reports on coplanar waveguide antenna and conductor-backed coplanar waveguide antenna.

Conductor-backed coplanar waveguide is a transmission structure similar to coplanar waveguide (CPW) but with a lower ground plane. In circuits design, CBCPW is different from CPW because they have different calculation formulas on the characteristic impedance. The ground plane of the CBCPW enhances the antenna's mechanical strength. It is noted that CPW/CBCPW are commonly used in RF integrated circuit (RFIC) and monolithic microwave integrated circuit (MMIC) because of the lower loss and good heat dissipation, and meanwhile, they do not need ground via holes. In this paper, a new coplanar waveguide rhomboid slot antenna with CBCPW feeding is developed to realize wideband, favorable gain and omnidirectional radiation characteristic in H plane. In order to show the advantages of the proposed antenna, circular and rectangular slot antennas based on CPW have been used for comparison, and better antenna performance such as wider bandwidth, higher gain, and even smaller antenna size have been verified for the proposed rhomboid slot antenna. The proposed CPW rhomboid slot antenna with CBCPW feeding has been fabricated and measured, and simple antenna structure, wide fractional bandwidth of 55.6 %, and a maximum gain of 6.2dBi have been obtained.

## 2 Antenna structure and construction

The slot antenna can be equivalent to magnon antenna because they have similar radiation principle. Since the E and H planes of the magnon are similar to the H and E planes of the electric cell, so their field distributions satisfy the duality principle. For the slot antenna, the electric field intensity E_s_ and magnetic field intensity Hs can be expressed as [19].

(1) $$\left\{ \begin{array}{l} E_{s}=j\frac{kI_{0}^{m}L}{8\pi r}\sin\;\theta e^{-ikr}\\ H_{s}=-j\frac{kI_{0}^{m}L}{8\pi r\eta }\sin\;\theta e^{-ikr} \end{array}\right.$$

with $I_{0}^{m}=2U_{0}$. Here U0 is the driving voltage, L is the slot length, k is the propagation constant with k=2π/λ, and η is the wave impedance with a value of 120 π. When L is less than or equal to a wavelength (λ), the current distributed on the dipole is positive; while when L is greater than or equal to a wavelength, the current distributed on the dipole is negative, resulting in the appearance of side lobes in the antenna pattern and affecting the radiation performance of the symmetrical dipole.

**Fig. 1:**
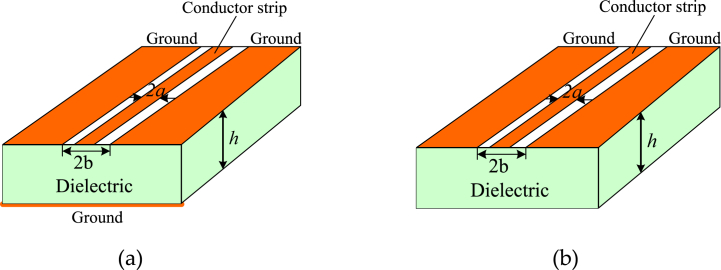
Structures of the conductor-backed coplanar waveguide and coplanar waveguide. (a) CBCPW. (b) CPW.

CBCPW and CPW structures are shown in Fig. 1(a) and (b), respectively. CBCPW is a transmission line that similar to the coplanar waveguide but with a lower ground plane, which may enhance the antenna mechanical strength. The conductor strip width of the CBCPW is 2a, and the total width of conductor strip and two etched slots is 2b. The substrate height of CBCPW is h. The characteristic impedance of the CBCPW is written as [20].

(2) Z=060πεeff1K(k)K(k3)K(k′)K(k3′)

where K is the complete elliptic integrals of the first kind, and the effective dielectric constant can be formulated as

(3) $$\varepsilon_{eff}=\frac{1+\varepsilon_{r}\frac{K\left(k^{\prime }\right)K\left(k_{3}\right)}{K\left(k\right)K\left(k_{3}^{\prime }\right)}}{1+\frac{K\left(k^{\prime }\right)K\left(k_{3}\right)}{K\left(k\right)K\left(k_{3}^{\prime }\right)}}$$

with

(4) $$\left\{ \begin{array}{l} k=\alpha /b\\ k_{3}=\tanh\left(\pi \alpha /2h\right)\tanh\left(\pi b/2h\right)\\ k^{\prime }=\sqrt{1.0-k^{2}}\\ k_{3}^{\prime }=\sqrt{1.0-k_{3}^{2}} \end{array}\right.$$

The characteristic impedance of the CPW can be expressed as [20].

(5) $$Z_{c}=\frac{30\pi }{\sqrt{\varepsilon_{re}}}\cdot \frac{K\left(k^{\prime }\right)}{K\left(k\right)}$$

with

(6) $$\varepsilon_{re}=1+\frac{\varepsilon_{r}-1}{2}∙\frac{K\left(k^{\prime }\right)}{K\left(k\right)}∙\frac{k\left(k_{1}\right)}{k\left(k_{1}^{\prime }\right)}$$

Here $k_{1}^{\prime }=\sqrt{1-k_{1}^{2}}$.

**Fig. 2:**
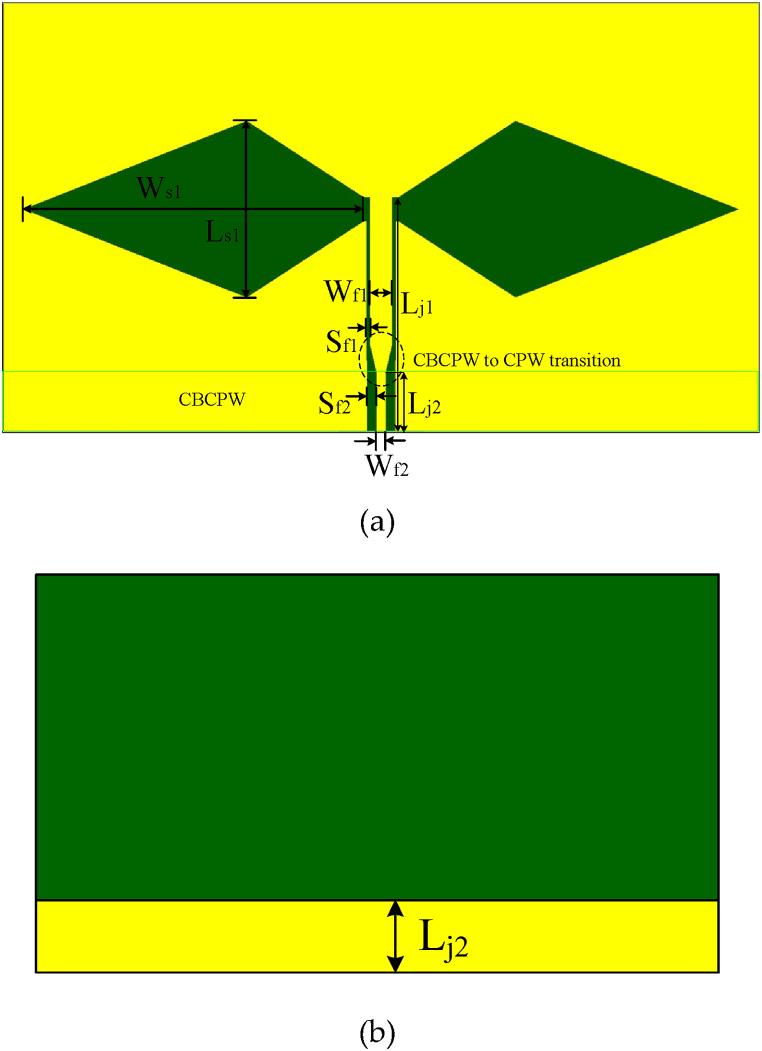
Structure of the wideband rhomboid slot antenna with CBCPW feeding.

**Fig. 3:**
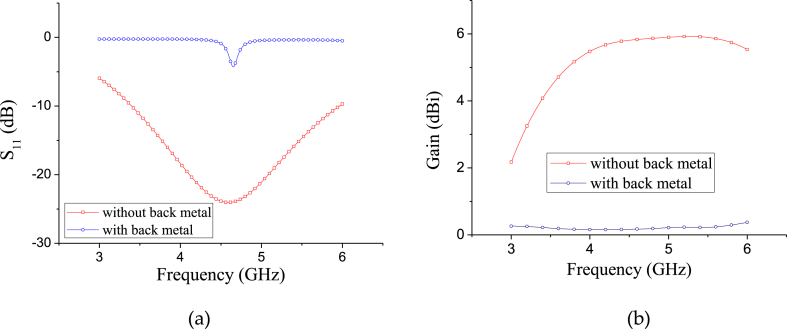
Simulated S11 and gain comparison of the rhomboid slot antenna with and without the back metal. (a) S11 comparison. (b) Gain comparison.

The proposed antenna is composed of a pair of rhomboid slots connected by the feeding line based on the coplanar waveguide and the conductor-backed coplanar waveguide feeding, as shown in Fig. 2(a) and (b), respectively. There is a transition between CBCPW and CPW because they have different characteristic impedance. In order to improve the radiation, major part metal on the backside has been removed. It is noted that lots of ground of the CPW can be used to etch multiple slots to introduce wide impedance bandwidth. When the current is fed into the antenna through feeding-source, it flows into the wide rhomboid slot and converts the CPW traveling wave into electromagnetic wave in space. When the working frequency is designed at 4.6 GHz with a relative impedance bandwidth of about 55.6 %, and a S11 attenuation of 24 dB, the dimensions of the CPW rhomboid slot antenna are obtained as Ls1 = 29 mm, Ws1 = 15 mm, Wf1 = 1.9 mm, Sf1 = 0.3 mm, Wf2 = 0.8 mm, Lj1 = 19.9 mm, and Lj2 = 5 mm. The antenna substrate is using Rogers 5880 which has a relative permittivity of 2.2. Simulated S11 and gain comparison of the rhomboid slot antenna with and without the back metal is shown in Fig. 3, here the case without back metal refers to that the ground metal on the back of the antenna is removed except for the CBCPW part. It is seen from the comparison results that both the matching and the gain of the rhomboid slot antenna have been importantly improved when the backside ground metal is removed.

**Fig. 4:**
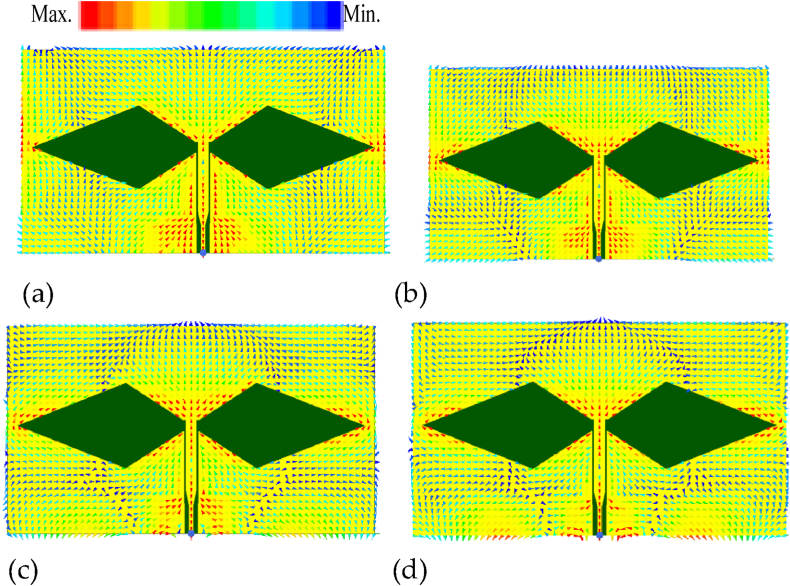
Simulated rhomboid slot antenna current distributions in different modes. (a) Mode 1 at 4.19 GHz. (b) Mode 2 at 4.731 GHz. (c) Mode 3 at 5.469 GHz. (d) Mode 4 at 5.767 GHz.

Modes of the rhomboid slot antenna are also researched, as shown in Fig. 4. Where, four modes have been excited in the antenna working bandwidth. Mode 1 is at 4.19 GHz, Mode 2 is at 4.731 GHz, Mode 3 is at 5.469 GHz, and Mode 4 is at 5.767 GHz, and their current distributions are shown in Fig. 4(a), (b), (c), and (d), respectively. Multiple modes would introduce a wideband.

## 3 Simulated antenna performance comparison

**Fig. 5:**
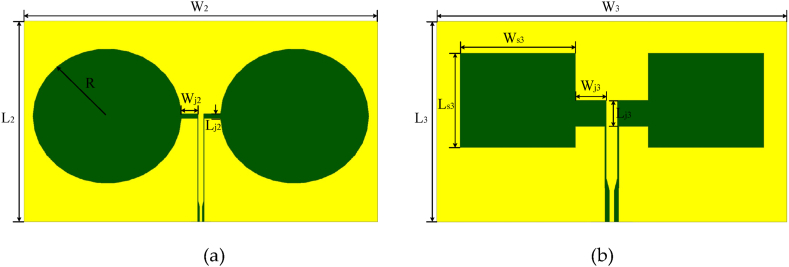
Circular slot antenna and rectangular slot antenna. (a) CPW circular slot antenna. (b) CPW rectangular slot antenna.

In order to show the advantages of the proposed CPW rhomboid slot antenna, CPW circular slot antenna and CPW rectangular slot antenna shown in Fig. 5(a) and (b), respectively, are used for comparison. The conductor strip width of the CBCPW feeding line for the three wide-slot antennas are equal, it is the same case for the narrow slot width of the feeding line. All of the CBCPW feeding lines have length of 5 mm as labeled by Lj2 in Fig. 2, and the slot antennas have the same substrate. In this research, it is also noticed that the rhomboid slot brings stronger current than the circular slot, which would introduce better radiation (better gain).

In order to compare the performance of CBCPW slot antennas with different shapes, additional circular wide-slot antenna and rectangular wide-slot antenna are also designed, as shown in Fig. 5(a) and (b), respectively. The dimension of the CPW circular slot antenna is obtained as W2 = 128.3 mm, L2 = 80.4 mm, R = 27 mm, Wj2 = 6 mm, Lj2 = 2 mm, while the dimension of the CBCPW rectangular slot antenna is obtained as W3 = 64.5 mm, L3 = 38.2 mm, Ws3 = 20 mm, Ls3 = 18 mm, Wj3 = 5.3 mm, Lj3 = 5 mm. The conductor strip width of the CBCPW feeding line for the three wide-slot antennas are equal, it is the same case for the narrow slot width of the feeding line. All of the CBCPW feeding lines have length of 5 mm as labeled by Lj2 in Fig. 2, and the slot antennas have the same substrate. In this research, it is also noticed that the rhomboid slot brings stronger current than the circular slot, which would introduce better radiation (better gain).

**Fig. 6:**
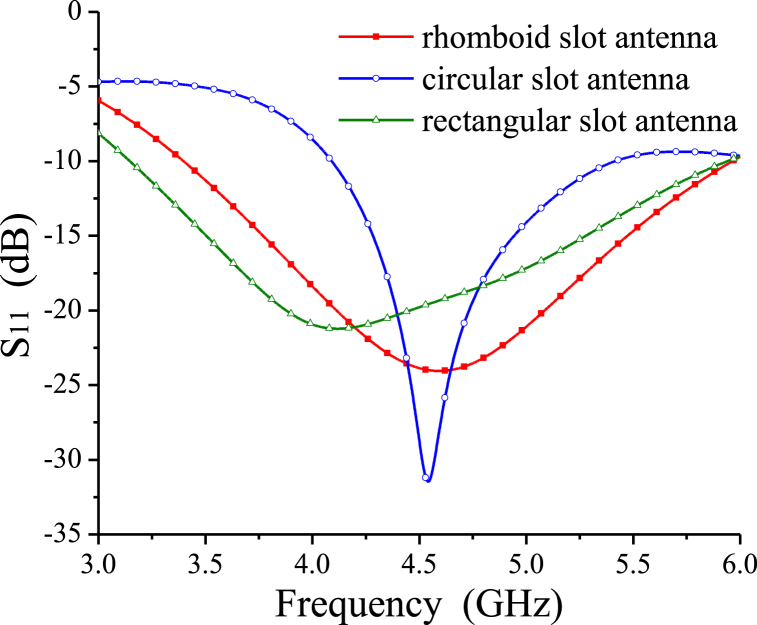
S-parameter results comparison for the rhomboid/circular/rectangular slot antennas. For the circular slot antenna, W2 = 128.3 mm, L2 = 80.4 mm, R = 27 mm, Wj2 = 6 mm, Lj2 = 2 mm. For the rectangular slot antenna, W3 = 64.5 mm, L3 = 38.2 mm, Ws3 = 20 mm, Ls3 = 18 mm, Wj3 = 5.3 mm, Lj3 = 5 mm.

**Fig. 7:**
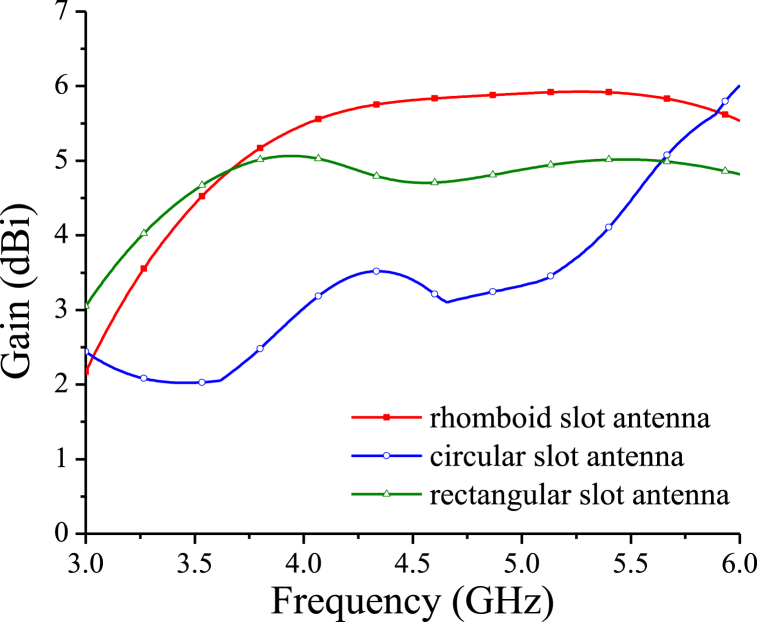
Gain comparison for the rhomboid/circular/rectangular slot antennas.

Simulated S-parameter and gain comparison of the CPW rhomboid/circular/rectangular slot antennas are shown in Fig. 6, Fig. 7, respectively. It is seen that in the same frequency band from 3 GHz to 6 GHz, bandwidth of the circular slot antenna is far less than that of the rhomboid and rectangular slot antennas, as shown in Fig. 6, while the rectangular slot antenna has a gain of no more than 5 dBi in 3–6 GHz, and circular slot antenna has smaller in-band gain, as shown in Fig. 7. It is also noted that for the similar circuit size, both of the rhomboid and rectangular slot antennas have wideband characteristics, but the average in-band gain of the rhomboid slot antenna is higher than that of the rectangular slot antenna, as shown in Fig. 7. The CPW rhomboid slot antenna has a fractional impedance bandwidth of about 55.6 %, and a maximum gain of 5.9 dBi.

**Fig. 8:**
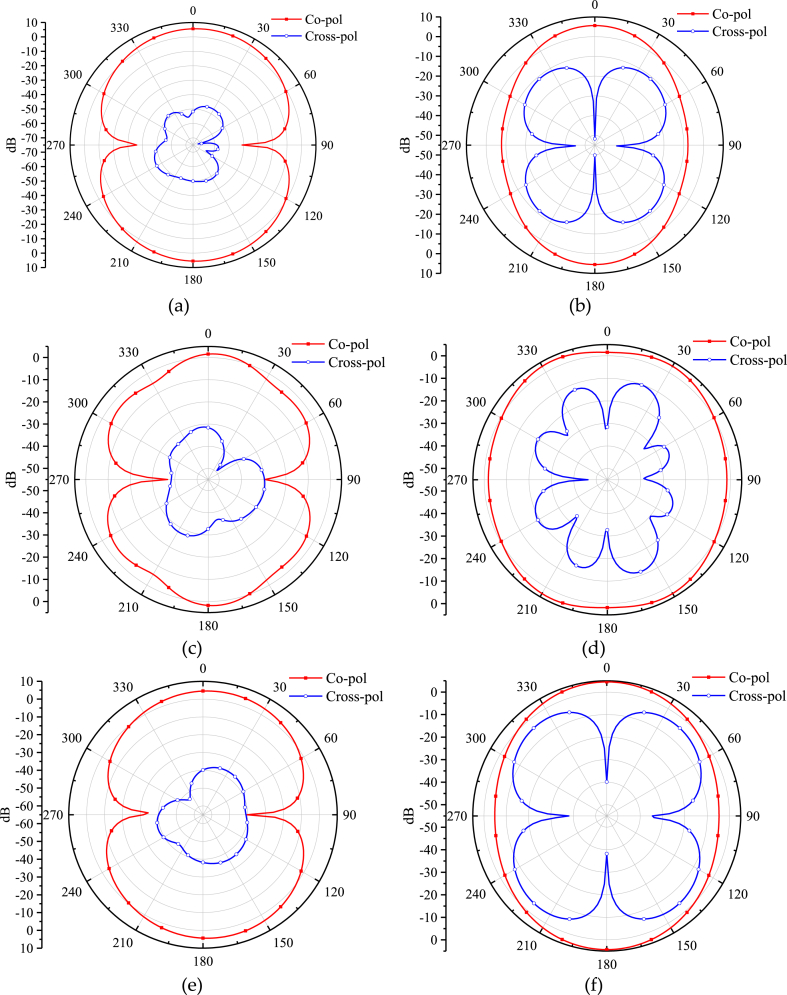
Radiation pattern of the rhomboid/circular/rectangular slot antennas at 4.6 GHz. (a) E-plane of the rhomboid slot antenna. (b) H-plane of the rhomboid slot antenna. (c) E-plane of the circular slot antenna. (d) H-plane of the circular slot antenna. (e) E-plane of the rectangular slot antenna. (f) H-plane of the rectangular slot antenna.

Simulated radiation pattern of the rhomboid/circular/rectangular slot antennas are shown in Fig. 8. It is seen that all of the CPW slot antennas have bidirectional radiation characteristics in the E plane, while they have approximately omnidirectional radiation characteristics in the H plane. It is also noticed from Fig. 8 that the radiation pattern of E plane of the circular slot antenna is distorted, and the cross polarization of the rhomboid slot antenna is lower than that of the rectangular slot antenna. So in summary, rhomboid slot antenna has better performance than that of the circular and rectangular slot antennas. The simulation is performed by using ANSYS HFSS.

## 4 Realization of the CPW rhomboid slot antenna

The design process of the proposed CPW rhomboid slot antenna is summarized as 1) calculate the width and slot gap of the feeding line when the working frequency, substrate material and thickness are determined; 2) design the transition part between CBCPW and CPW; 3) determine the shape and dimension of the rhomboid slot according to the working frequency and impedance bandwidth; 4) antenna simulation and optimization by using HFSS.

**Fig. 9:**
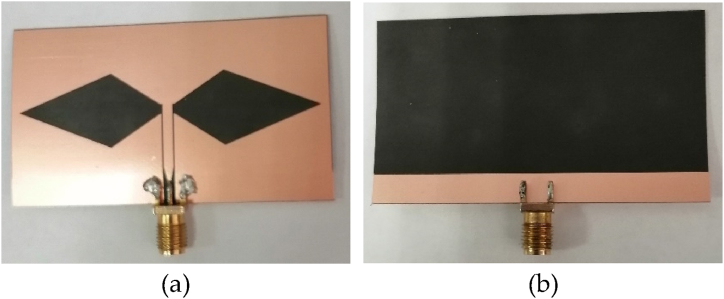
Manufactured CPW rhomboid slot antenna with CBCPW feeding. (a) Top view. (b) Back view.

**Fig. 10:**
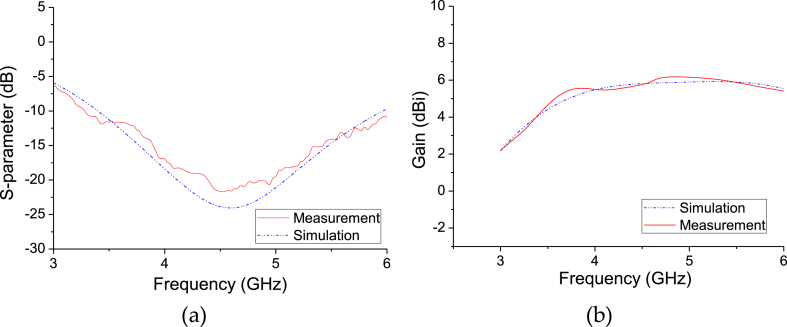
Experimental results of S-parameter and gain for the CPW rhomboid slot antenna. (a) Measured S11. (b) Measured antenna gain.

**Fig. 11:**
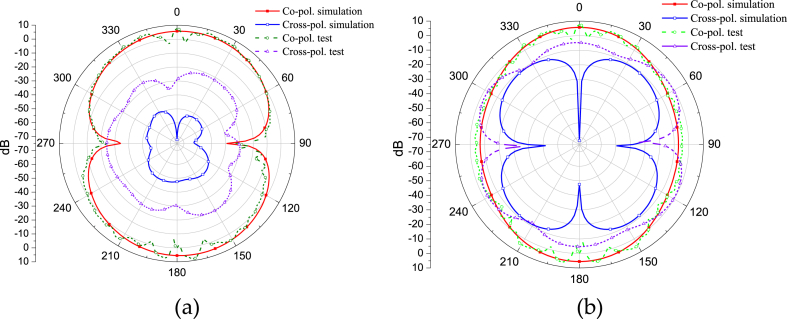
Experimental patterns for the CPW rhomboid slot antenna at 4.6 GHz. (a) Measured co-polarization and cross-polarization patterns of the E plane. (b) Measured co-polarization and cross-polarization patterns of the H plane. Here pol. represents polarization.

The proposed CPW rhomboid slot antenna with CBCPW feeding has been manufactured, as shown in Fig. 9. Where the antenna top view and the back view are shown in Fig. 9(a) and (b), respectively. The experimental results for S-parameter and antenna gain are shown in Fig. 10(a) and (b), respectively. It is seen that experimental results approach to the simulated results. Measured results verify that the CPW rhomboid slot antenna works at 4.6 GHz, and it has a relative impedance bandwidth of about 55.6 %, and a gain of more than 5.5 dBi from 4 GHz to 6 GHz. Measured co-polarization and cross-polarization patterns of the E/H planes for the CPW rhomboid slot antenna are shown in Fig. 11(a) and (b), respectively. It is seen that omnidirectional radiation characteristic is obtained, and the tested results of co-polarization are similar to the simulated results, while the tested results of cross-polarization have some errors with the simulated ones. The simulation/measurement difference is maybe due to manufacture error.

**Fig. 12:**
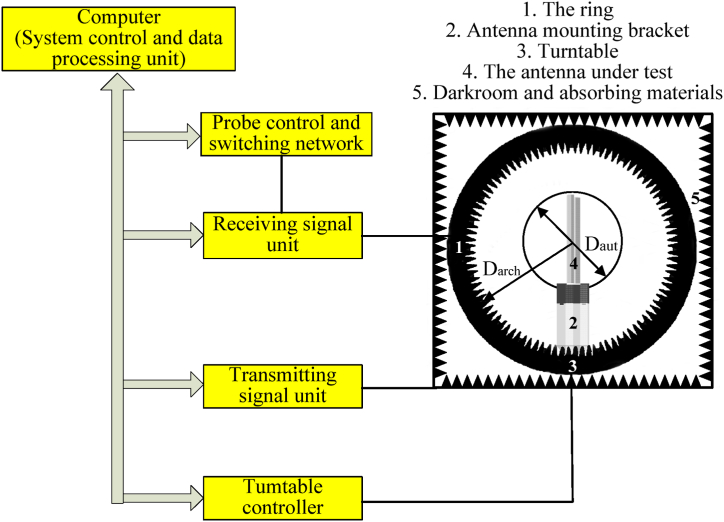
The construction of the spherical multi-probe test system.

Measured S11 of the antenna is performed by Agilent E5071C vector network analyzer, while the measured gain, radiation patterns and axial ratio of the antenna are achieved by spherical multi-probe test system which is composed of turntable controller, transmitting signal unit, receiving signal unit, probe control and switching network, system control and data processing unit, and darkroom which includes the ring, antenna mounting bracket, absorbing materials and the antenna under test, as shown in Fig. 12. Where Daut is the maximum caliber of the antenna under test, and Darch is the probe array torus bore diameter. The test procedure can be summarized as.

1) Gain calibration by using standard gain horn.
2) Set up the antenna under test.
3) Open the test software, and set the test frequency and test port.
4) Use test software for automatic testing and save test data.
5) The test data of the standard gain antenna and the antenna to be measured are imported into the data processing software to obtain the gain and radiation pattern parameters.

Comparison of this work and the related reports is shown in Table 1. It is seen that compared with some reported slot antennas, our proposed CPW rhomboid slot antenna has wider bandwidth and higher gain than references [9,[12], [13], [14], [15]], and smaller profile has also been obtained. On the other hand, our design is using CPW structure, which is different from the commonly used microstrip structure. The proposed CPW antenna is not only superior to the related microstrip ones, but also has potential applications in millimeter waveband because CPW has favorable performance in that band.

**Table 1:** Comparison of this work and some related slot antennas.

**Reference**	**Center frequency** (GHz)	**Profile (λ**_**0**_**)**	**Fractional bandwidth**	**Maximum gain (dBi)**	**Antenna structure**
[9]	4.85/3.6–6.1	0.28	51.5 %	4	Microstrip
[12]	4.3/3.13–5.53	0.024	50 %	2.35	Microstrip
[13]	3.598/3.48–3.72	Not shown	5.4 %	3.63	Microstrip
[14]	4.5/3.4–5.6	0.14	45 %	2	Microstrip
[15]	2.2/1.7–2.7	0.011	46 %	3.4	Microstrip
This work	4.6/3.32–5.88	0.005	55.6 %	6.2	CPW

## 5 Conclusion

According to the advantages of the CPW/CBCPW and their adequate ground area, a new CPW wideband rhomboid slot antenna with a pair of rhomboid slots on the ground by using CBCPW feeding is designed, and the proposed antenna performance has been compared with CPW circular/rectangular slot antennas. While the proposed CPW rhomboid slot antenna has also been compared with some related reports, and the advantages of wide bandwidth, low profile and favorable gain have been verified. The CPW rhomboid slot antenna has been manufactured and measured, and its wide relative bandwidth of about 55.6 % and a maximum gain of 6.2 dBi have been verified. Compared with the commonly used microstrip antennas, the coplanar waveguide antenna not only has wider bandwidth, but also has potential applications in higher frequency band such as millimeter waveband.

## CRediT authorship contribution statement

**Yulei Xue:** Software, Project administration, Methodology, Funding acquisition. **Ruiqiang Yan:** Validation, Supervision, Data curation, Conceptualization. **Weiping Huang:** Resources, Methodology. **Yanchen Dong:** Voluntarily renounce authorship of this manuscript.

## Informed consent statement

Informed consent was obtained from all subjects involved in the study.

## Data availability statement

The data used to support the findings of this study are available from the corresponding author upon request.

## Funding

This research was funded by the Beidou New Time-space Intelligent Industry Development Collaborative Innovation Center 2021YFB1407001.

## Declaration of competing interest

The authors declare that they have no known competing financial interests or personal relationships that could have appeared to influence the work reported in this paper.
